# Mitochondrial Dynamics and Mitochondria-Lysosome Contacts in Neurogenetic Diseases

**DOI:** 10.3389/fnins.2022.784880

**Published:** 2022-01-31

**Authors:** Jordi Pijuan, Lara Cantarero, Daniel Natera-de Benito, Arola Altimir, Anna Altisent-Huguet, Yaiza Díaz-Osorio, Laura Carrera-García, Jessica Expósito-Escudero, Carlos Ortez, Andrés Nascimento, Janet Hoenicka, Francesc Palau

**Affiliations:** ^1^Laboratory of Neurogenetics and Molecular Medicine – IPER, Institut de Recerca Sant Joan de Déu, Barcelona, Spain; ^2^Centro de Investigación Biomédica en Red de Enfermedades Raras (CIBERER), Barcelona, Spain; ^3^Neuromuscular Unit, Department of Pediatric Neurology, Hospital Sant Joan de Déu, Barcelona, Spain; ^4^Department of Genetic Medicine – IPER, Hospital Sant Joan de Déu, Barcelona, Spain; ^5^Clinic Institute of Medicine and Dermatology (ICMiD), Hospital Clínic, Barcelona, Spain; ^6^Division of Pediatrics, Faculty of Medicine and Health Sciences, University of Barcelona, Barcelona, Spain

**Keywords:** mitochondrial dynamics, membrane contact sites (MCSs), mitochondria, lysosome, neurogenetic diseases

## Abstract

Mitochondrial network is constantly in a dynamic and regulated balance of fusion and fission processes, which is known as mitochondrial dynamics. Mitochondria make physical contacts with almost every other membrane in the cell thus impacting cellular functions. Mutations in mitochondrial dynamics genes are known to cause neurogenetic diseases. To better understand the consequences on the cellular phenotype and pathophysiology of neurogenetic diseases associated with defective mitochondrial dynamics, we have compared the fibroblasts phenotypes of (i) patients carrying pathogenic variants in genes involved in mitochondrial dynamics such as *DRP1* (also known as *DNM1L*), *GDAP1*, *OPA1*, and *MFN2*, and (ii) patients carrying mutated genes that their dysfunction affects mitochondria or induces a mitochondrial phenotype, but that are not directly involved in mitochondrial dynamic network, such as *FXN* (encoding frataxin, located in the mitochondrial matrix), *MED13* (hyperfission phenotype), and *CHKB* (enlarged mitochondria phenotype). We identified mitochondrial network alterations in all patients’ fibroblasts except for *CHKB*^Q198*/Q198*^. Functionally, all fibroblasts showed mitochondrial oxidative stress, without membrane potential abnormalities. The lysosomal area and distribution were abnormal in *GDAP1*^W67L/W67L^, *DRP1*^K75E/+^, *OPA1*^F570L/+^, and *FXN*^R165C/GAA^ fibroblasts. These lysosomal alterations correlated with mitochondria-lysosome membrane contact sites (MCSs) defects in *GDAP1*^W67L/W67L^ exclusively. The study of mitochondrial contacts in all samples further revealed a significant decrease in *MFN2*^R104W/+^ fibroblasts. GDAP1 and MFN2 are outer mitochondrial membrane (OMM) proteins and both are related to Charcot-Marie Tooth neuropathy. Here we identified their constitutive interaction as well as MFN2 interaction with LAMP-1. Therefore MFN2 is a new mitochondria-lysosome MCSs protein. Interestingly, *GDAP1*^W67L/W67L^ and *MFN2*^R104W/+^ fibroblasts carry pathogenic changes that occur in their catalytic domains thus suggesting a functional role of GDAP1 and MFN2 in mitochondria–lysosome MCSs. Finally, we observed starvation-induced autophagy alterations in *DRP1*^K75E/+^, *GDAP1*^W67L/W67L^, *OPA1*^F570L/+^, *MFN2*^R104W/+^, and *CHKB*^Q198*/Q198*^ fibroblasts. These genes are related to mitochondrial membrane structure or lipid composition, which would associate the OMM with starvation-induced autophagy. In conclusion, the study of mitochondrial dynamics and mitochondria-lysosome axis in a group of patients with different neurogenetic diseases has deciphered common and unique cellular phenotypes of degrading and non-degrading pathways that shed light on pathophysiological events, new biomarkers and pharmacological targets for these disorders.

## Introduction

Mitochondria are key organelles that produce cellular ATP and are also involved in the cell metabolic status, programmed cell death, calcium homeostasis, and the generation and control of reactive oxygen species (ROS) (Wai and Langer, 2016). They are highly dynamic structures, which change their morphology and spatial distribution according to metabolic requirements, allowing cells to adapt to energy demands and maintain homeostasis (Kyriakoudi et al., 2021). This changeable and adaptable nature, known as mitochondrial dynamics, includes both the regulation of mitochondrial architecture, mediated by the fusion and fission, and the movement of mitochondria along the cytoskeleton (Chan, 2006).

Mitochondria can be found as isolated organelles or forming larger networks. In mammals, mitochondrial fusion is regulated by the large GTPases mitofusin 1 (MFN1) and mitofusin 2 (MFN2) of the outer mitochondrial membrane (OMM), and optic atrophy 1 (OPA1) of the inner mitochondrial membrane (IMM) (Cipolat et al., 2004). Mitochondrial fission is carried out by dynamin-related protein 1 (DRP1), which translocates from the cytosol to mitochondria, binds to its OMM partners [mitochondrial fission factor (MFF)], mitochondrial dynamics protein of 49 and 51 kDa (MID49 and MID51), and mitochondrial fission 1 protein (FIS1) and drives the scission (Otera et al., 2010). In addition, GDAP1 has also been proposed to participate in the mitochondrial fission process (Niemann et al., 2005). Disruption of mitochondrial fusion/fission equilibrium results in a fragmented or elongated mitochondrial network that has been associated with various pathological conditions (Chan, 2006, 2020).

Outer mitochondrial membranes make physical contact with virtually all other cell membranes, not only regulating mitochondrial function but also impacting larger inter-organelle networks and cellular functions (Lackner, 2019; Giacomello et al., 2020). Communication and contacts between mitochondria and endoplasmic reticulum at mitochondria-associated membranes (MAMs) have been extensively studied (Krols et al., 2016). More recently, other mitochondrial membrane contact sites (MCSs) have been described for lysosomes (Wong et al., 2018; Cantarero et al., 2020) and peroxisomes (Fan et al., 2016). Since the number and function of mitochondrial MCSs have increased in recent years (Silva et al., 2020), their characterization is leading to the emerging understanding of mitochondrial dynamics dysfunction. An increasing number of genes related to the mitochondrial network function and dynamics have been associated with several Mendelian disorders in humans, showing the sensitivity and responsiveness of mitochondrial membrane to cellular states (Chan, 2020; Giacomello et al., 2020; Yang et al., 2021). However, no study has focused on comparing the cellular impact of these conditions on mitochondrial dynamics in patients.

To better understand the pathophysiology of mitochondrial dynamics-associated diseases, we studied the dysfunctional effects in the mitochondrial biology and phenotypes in fibroblasts from patients with disorders caused by mutations in nuclear genes associated with mitochondrial dynamics in comparison with neurodegenerative disorders that have changes in mitochondria but are not related to the mitochondrial network dynamics. We intend to identify common cellular phenotypes related to mitochondrial pathophysiology, new biomarkers and pharmacological targets. Our results show that the study of mitochondria-lysosome axis and mitochondria-lysosome contacts are a useful tool to characterize pathophysiological events of degradative and non-degradative pathways in neurogenetic diseases.

## Materials and Methods

### Ethical Considerations

All procedures complied with the ethical guidelines of Sant Joan de Déu Children’s Hospital and were approved by the Clinical Research Ethics Committee under reference PIC-223-19. Informed consent was obtained from the patients, the patient’s parents, or legal guardians.

### Bioinformatic Analyses

The pathogenicity classification of genetic variants was done using VarSome^1^ (*last accessed March 24, 2021*) and the American College of Medical Genetics and Genomics (ACMG) standard guidelines. Genome Aggregation Database (gnomAD; *last accessed March 24, 2021*)^2^ and CIBERER-Spanish Variant Server (CSVS; *last accessed March 24, 2021*)^3^ were used to evaluate the frequencies of the variants in global and Spanish populations, respectively. *In silico* analyses of the genetic variants were performed using: PROVEAN (Choi and Chan, 2015), FATHMM (Shihab et al., 2013), DANN (Quang et al., 2015), MutationTaster (Schwarz et al., 2014), and CADD (Rentzsch et al., 2019).

### Fibroblasts Culture

Skin biopsy was taken from the patient following standard clinical procedures (Zuber, 2002; Vangipuram et al., 2013). Healthy control fibroblasts were obtained from Sant Joan de Déu Children’s Hospital Biobank. Fibroblasts were cultured in Dulbecco’s Modified Eagles’ Medium high-glucose (Sigma-Aldrich, St. Louis, MO, United States) supplemented with 10% fetal bovine serum (FBS; Sigma-Aldrich), 2 mM L-glutamine (Sigma-Aldrich) and 100 mg/ml penicillin-streptomycin (Sigma-Aldrich) at 37°C in a 5% CO_2_ incubator. Cells were periodically tested for mycoplasma infection.

#### Drugs and Treatments

For the induction of autophagy by amino acid starvation, fibroblasts were washed three times with PBS and cultured in Earle’s Balanced Salt Solution (EBSS, Thermo Fisher Scientific, Waltham, MA, United States) for 4 h at 37°C. The specific inhibitor of vacuolar-type H^+^-ATPase Bafilomycin A1 (Sigma-Aldrich) was used at 200 nM for 4 h at 37°C.

### Immunofluorescence

Fibroblasts (3 × 10^4^) were seeded onto glass coverslips for 24 h, washed with phosphate buffer saline (PBS) and fixed in pre-warmed 4% paraformaldehyde (PFA) for 20 mins at room temperature. Fibroblasts were permeabilized with 0.2% Triton X-100 in PBS for 30 mins at room temperature and they were blocked with 1% bovine serum albumin (BSA) and 4% serum in PBS for 1 h at room temperature. The specific primary antibodies α-TOM20 mouse monoclonal (1:100; BD Transduction Laboratories, Franklin Lakes, NJ, United States; 612278), α-LAMP-1 mouse antibody (1:200; DSHB, Iowa, IA, United States; H4A3), α-LAMP-1 rabbit polyclonal (1:500; Abcam; ab24170) and α-p62/SQSTM1 rabbit polyclonal (1:200; Sigma-Aldrich; P0067) were incubated overnight at 4°C. Primary antibodies were visualized using Alexa Fluor^®^ 488 or Alexa Fluor^®^ 633-labeled secondary antibody (1:500; Thermo Fisher Scientific; A11034, A11029, and A21070). After 2 h of incubation, fibroblasts were rinsed with PBS and mounted on a coverslip using Fluoromont-G with DAPI (4′,6-diamidino-2-phenylindole) (Thermo Fisher Scientific).

### *In situ* Proximity Ligation Assay

Fibroblasts (3 × 10^4^) were seeded onto glass coverslips for 24 h, washed in PBS, fixed in pre-warmed 4% PFA for 20 mins at room temperature and permeabilized with ice-cold methanol at −20°C for 20 mins. After 1 h of incubation at 37°C with the blocking solution in a pre-heated humidity chamber, fibroblasts were incubated overnight at 4°C with the specific primary antibodies: α-GDAP1 mouse monoclonal (1:200, Abcam, Cambridge, United Kingdom; ab194493), α-MFN2 mouse monoclonal (1:100, Abcam; ab56889) and α-LAMP-1 rabbit polyclonal (1:100, Abcam; ab24170). Afterward, we perform the PLA assay according to the manufacturer’s instructions (Duolink^®^ *In Situ* Detection Red Starter [Mouse/Rabbit] Kit; Sigma-Aldrich) and the coverslips were mounted with Duolink *In situ* Mounting Medium with DAPI. Images were acquired with a Leica TCS SP8 X White Light Laser confocal microscope using 63× oil immersion objective and Z-stacks were acquired every 0.1 μm along with the cell thickness. For PLA with co-stained mitochondria and lysosomes, images were acquired using 100× oil immersion objective and deconvolution was performed with Huygens Essential software v 4.4 0p6 (SVI, Leiden, Netherlands). Image analysis was performed using maximum intensity projection in Image J/Fiji software (NIH, US National Institutes of Health, Bethesda, MD, United States). For each antibody, a negative control experiment was performed, where only one antibody was incubated with the PLA probes.

### Mitochondrial Oxidative Stress and Mitochondrial Membrane Potential

We analyzed the mitochondrial membrane potential and the mitochondrial oxidative stress with TMRM and MitoSOX probes, respectively. We measured these parameters using two different approaches: live-cell imaging and flow cytometry.

#### Live Cell Imaging

Fibroblasts (1 × 10^5^) were seeded onto a glass coverslip and cultured for 24 h. Cells were washed with warmed PBS and loaded with 2.5 μM MitoSOX Red (Thermo Fisher Scientific) for 10 mins or 100 nM TMRM (Thermo Fisher Scientific) for 30 mins in the dark at 37°C in a 5% CO_2_ incubator. *In vivo* images of cells were captured using a Leica TCS SP8 X White Light Laser confocal microscope (Leica Microsystems). The excitation/emission of TMRM and MitoSOX were detected in live-cell imaging through 552/574 and 510/580 nm wavelengths, respectively. As positive controls, fibroblasts were treated with 2 mM H_2_O_2_ (Sigma-Aldrich) for 5 mins to induce oxidative stress or 50 μM FCCP (Sigma-Aldrich) for 15 mins to depolarize the mitochondrial membrane.

#### Flow Cytometry

Fibroblasts were grown in 12-well plates (1 × 10^5^ cells/well) for 48 h, and then they were trypsinized, washed with PBS and resuspended in 5 μM MitoSOX Red (Thermo Fisher Scientific) for 15 mins or 100 nM Tetramethylrhodamine Methyl Ester Perchlorate (TMRM; Thermo Fisher Scientific; T668) and 100 nM MitoTracker Green (MTG; Invitrogene; M7514) for 30 mins in the dark at 37°C. After that fibroblasts were acquired on ACEA NovoCyte 3000 Flow Cytometer (ACEA Biosciences, Biosciences, San Diego, CA, United States). For each assay, 10,000 events were collected and analyzed. Fibroblasts were treated with 500 μM hydrogen peroxide (H_2_O_2_) (Sigma-Aldrich) for 15 mins or 50 μM Carbonyl cyanide 4-(trifluoromethoxy) phenylhydrazone (FCCP; Sigma-Aldrich, C2920) for 5 mins at 37°C as positive controls for mitochondrial oxidative stress and mitochondrial membrane potential, respectively. Final values for MitoSOX and TMRM/MTG fluorescence were calculated as relative fluorescence values over the mean of two independent healthy controls.

### Western Blot and Co-immunoprecipitation Assays

Fibroblasts were homogenized in lysis buffer (50 mM Tris HCl pH 7.4, 1.5 mM MgCl_2_, 5 mM EDTA, 1% Triton X-100, 50 mM NaF, and 1 mM Na_2_VO_3_) containing a protease inhibitor cocktail (Complete Mini-Protease Inhibitor Cocktail, Roche, Branchburg, NJ, United States). Homogenates were centrifuged at 13,200 rpm for 15 min at 4°C and the protein concentration of the supernatant was quantified by the BCA method (Thermo Fisher Scientific). For immunoprecipitation assays, 1 mg of total lysate was incubated with the specific antibody for 6–8 h at 4°C followed by incubation with Protein G Sepharose*™* 4 Fast Flow (GE, Healthcare) overnight at 4°C. Beads were softly washed with lysis buffer, resuspended in Laemmli Buffer, and heated at 95°C. Lysates and Co-immunoprecipitation (co-IPs) were resolved in sodium dodecyl sulfate-polyacrylamide gels (SDS-Page) and transferred onto Amersham Hybond PVDF membranes (GE Healthcare, Chicago, IL, United States). The membranes were blocked with 5% defatted-milk in TBS-0.1% Tween 20 buffer (25 mM Tris, 50 mM NaCl, 2.5 mM KCl, and 0.1% Tween-20). Afterward, the membranes were blotted with the specific primary antibodies α-MFN2 rabbit polyclonal (1:500; Sigma-Aldrich; M6319), α-MFN2 mouse monoclonal (1:1,000; Abcam; ab56889), α-GDAP1 rabbit polyclonal (1:500; HPA024334; Sigma-Aldrich), α-LAMP-1 rabbit polyclonal (1:500; Sigma-Aldrich; L1418), α-LAMP-1 mouse monoclonal (1:500; DSHB; H4A3), α-p62/SQSTM1 rabbit polyclonal (1:500; Sigma-Aldrich; P0067), α-LC3B rabbit polyclonal (1:500; Sigma-Aldrich, L7543), and α-β Actin mouse monoclonal (1:8,000 5% BSA; Sigma-Aldrich; A5316) which were detected using secondary antibodies coupled to horseradish peroxidase. Proteins were processed for chemiluminescence with Amersham ECL Prime Western Blotting Detection Reagent and visualized by iBright*™* CL1000 Imaging System (Thermo Fisher Scientific).

### Image Acquisition and Analyses

Super-resolution images were acquired with a Leica TCS SP8 X White Light Laser confocal microscope with Hybrid spectral detectors and HyVolution (Leica Microsystems, Wetzlar, Germany) using the Leica LAS X software (version 3.1.5). Images were acquired using 100× or 63× oil immersion objectives. The original data was stored as 16-bit greyscale images with a spatial resolution of 1,024 × 1,024 pixels. Z-stacks were acquired every 0.16 μm along with the cell thickness. Image processing and analysis were performed using Image J/Fiji software (NIH, US National Institutes of Health) and the Leica Application Suite X (LAS-X) software (Leica Microsystems). To compare the data, identical settings were used for image acquisition of different experiments and negative control samples were used for background setting previous to image acquisition.

#### Mitochondrial Network Morphology

Mitochondrial network morphology was analyzed by Mito-morphology macro with ImageJ/Fiji software (NIH) (Dagda et al., 2009).

#### Lysosome Morphology

The morphology of lysosomes was assessed using ImageJ/Fiji (NIH). First, maximum intensity projections were generated from Z-stacks followed by automated 8-bit Otsu-thresholding. Lastly, the binary images were evaluated to obtain the total area of LAMP-1 in each cell.

#### Autophagic Flux (p62)

The number of aggregates of p62 was evaluated in ImageJ/Fiji (NIH). Three intermediate Z-stacks were projected and an automated Otsu-thresholding was applied to 8 bit-images before the Particle Analysis.

#### Living Cell Acquisition and Analyses (Tetramethylrhodamine Methyl Ester Perchlorate and MitoSOX)

Z-stacks were acquired every 1 μm along with the cell thickness to avoid photo-bleaching. The total fluorescent intensity signal per cell was measured after the generation of maximum intensity projections, followed by automated 8-bit Otsu-thresholding in ImageJ/Fiji (NIH). The intensity color maps were performed in LAS-X software (Leica Microsystems) using a spectrum intensity map.

### Statistical Analyses

All data are expressed as mean ± standard deviation (SD) or box plots showing the median, box edges represent the [25th and 75th percentiles], and the whiskers extend to the minimum and maximum values. The normality of data was assessed by the Kolmogorov-Smirnov test. Statistical analysis was performed using GraphPad Prism (version 8.0.1; GraphPad Software, Inc., La Jolla, CA, United States) with a minimum of three independent experiments. The specific test applied in each case is indicated in the figure legend. *P*-values less than 0.05 were considered significant. *P*-values are indicated by asterisks *P < 0.05, ^**^P < 0.01, ^***^P < 0.001.

## Results

### Clinical and Genetic Features of Patients

**FIGURE 1:**
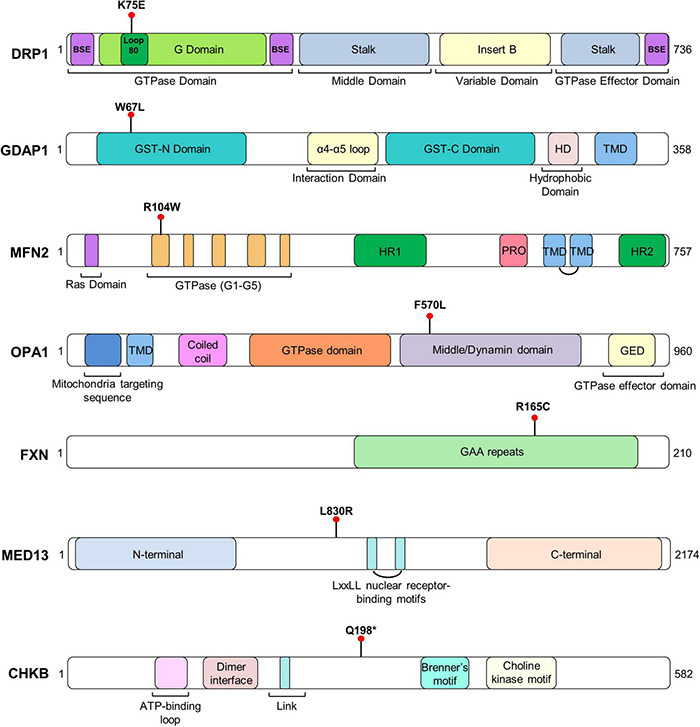
Representative scheme of the domains and structure of each protein with the location of patients’ variants. TM, transmembrane domain.

To discover cellular markers related to the effect of pathogenic variants in neurogenetic diseases, we included seven patients affected by mutations of different Mendelian disorders that are associated with mitochondrial phenotypes. We ascertained the patients based on two categories: (i) four involving genes related to mitochondrial network dynamics, either the fission process, *DRP1* (also *DNM1L*, encoding dynamin-related protein 1) and *GDAP1* (ganglioside induced differentiation-associated protein 1) or the fusion process, *OPA1* (optic atrophy protein 1) and *MFN2* (mitofusin 2), and (ii) three whose the gene dysfunction affects mitochondria or induces a mitochondrial phenotype but are not directly involved in mitochondrial dynamics, *FXN* (frataxin, mitochondrial matrix) (Lynch and Farmer, 2021), *MED13* (mediator complex subunit 13, a transcriptional coactivator that prevents mitochondrial fission and programmed cell death) (Cooper et al., 2014; Khakhina et al., 2014) and *CHKB* (choline kinase beta, phospholipids synthesis) (Mitsuhashi et al., 2011). We provided the clinical and genetic features (Table 1), the *in silico* analysis of the genetics variants (Table 2) and their location in the encoded proteins (Figure 1).

**TABLE 1:** Summary of the clinical and genetic features in the patients of this study.

Sex	Age at last visit (years)	Genetics: gene/variant	Age at onset (symptoms)	Clinical phenotype	Neurophysiological findings	Histopathological findings	Neuroimaging
M	8	*DRP1*: c.223A > G; p.K75E	10 months (motor delay)	Progressive spastic paraparesis with pyramidal signs in lower limbs, hyperreflexia and spastic gait. Slight elevation of long-chain fatty acids and delayed conduction of visually evoked potentials. CK: 240 (62-235)	NA	NA	Brain and medular MRI: Normal
F	30	*GDAP1*: (homozygous) c.200G > T; p.W67L	18 months (frequent falls)	Charcot-Marie-Tooth disease, axonal, type 2K (#607831). Distal weakness, areflexia, *pes cavus* and sensory ataxia. She became wheel chair-dependent at the age of 17 years old	Axonal sensory and motor neuropathy pattern	NA	Cranial CT: Normal
F	11	*OPA1*: c.1710T > G; p.F570L	8 months (congenital nystagmus)	Optic atrophy plus syndrome (#125250) Optic atrophy, sensorineural hearing loss, sensory ataxia, intellectual disability	Axonal sensory and motor neuropathy pattern	Accumulation of lipid droplets and mosaic pattern of COX-negative fibers	Brain and medular MRI: Normal
M	22	*MFN2*: c.310C > T; p.R104W	16 months (motor delay and falls)	Charcot-Marie-Tooth disease, axonal, type 2A2A (#609260). Progressive dystal weakness in lower and upper limbs. Areflexia. Muscle atrophy in distal parts, including feet deformities. He became wheelchair-dependent at the age of 12 years old. Optic neuropathy and vocal cord paralysis	Axonal sensory and motor neuropathy pattern	NA	Brain and medular MRI: Normal
M	44	*FXN*: c. 493C > T; p.R165C/GAA expansion	39	Friedreich ataxia, late onset variant. Trunk and limbs ataxia	Axonal sensory neuropathy	NA	NA
M	8 (died)	*MED13*: c.2489T > G; p.L830R	15 days (hypotonia)	Refractory epilepsy of neonatal onset, spastic tetraparesis (axial hypotonia and limb spasticity), pigmentary retinopathy, microcephaly, intellectual disability with absent speech, dysmorphic facial features, retrognathia, hypertelorism, flat philtrum and nystagmus. Hyperlactacidemia and metabolic acidosis, delayed myelination, trichorrhexis nodosa	Normal at 2 years old	Subsarcolemmal mitochondrial aggregates in some fibers as well as COX staining markedly reduced in others	Brain MRI at 2 years old: delayed myelination, decreased white matter volume and corpus callosum thinning
F	17	*CHKB:* (homozygous) c.592_593delCA; p.Q198*	12 months (global psychomotor delay and increased creatinkinase)	Congenital muscular dystrophy, megaconial type (#602541). Moderate intellectual disability and progressive proximal weakness. Wheelchair-dependent since she was 15 years old. Dilated cardiomyopathy. Behavioral disorder	Myopathic pattern	Dystrophic changes with necrosis and regeneration in muscle. Mitochondrial depletion in the center of the sarcoplasm as well as markedly enlarged mitochondria at the periphery of fibers (megaconial appearance)	Brain MRI: Normal

*M, male; F, female; NA, not available; MRI, magnetic resonance imaging.*

**TABLE 2:** *In silico* analysis of patients’ genetic variants.

Gene (Ref. sequence)	Nucleotide (amino acid) change	gnomAD/CSVS frequency	Segregation		Predictors of pathogenicity					

				Variants classification (ACMG)	FATHMM (score)	PROVEAN (score)	Mutation taster (*P*-value)	DANN (score)	CADD (score)	gnomAD (pLI/missense variation) (score)
*DNM1L (DRP1)* (NM_012062.3)	c.223A > G (p.K75E)	NR/NR	*de novo*	LP	Tolerated (−1.35)	Neutral (−1.08)	Benign (0.99)	Tolerated (0.97)	18.13	03.83
*GDAP1* (NM_018972)	c.200G > T (p.W67L)	NR/NR	M: Het F: Het	LP	Tolerated (−0.02)	Damaging (−10.85)	Disease causing (1)	Pathogenic (0.98)	32	0 1
*OPA1* (NM_130834)	c.1710T > G (p.F570L)	NR/NR	*de novo*	P	Damaging (−3.33)	Damaging (−5.57)	Disease causing (1)	Pathogenic (0.99)	25.1	0.99 1.97
*MFN2* (NM_0011276660.1)	c.310C > T (p.R104W)	NR/NR	*de novo*	P	Damaging (−4.18)	Damaging (−7.36)	Disease causing (1)	Pathogenic (0.99)	27.7	0.99 1.66
*FXN* (NM_000144.5)	c. 493C > T (p.R165C)	NR/NR	–	LP	Damaging (−4.51)	Damaging (−7.35)	Disease causing (1)	Pathogenic (0.99)	32	0.34 0.28
*MED13* (NM_005121.2)	c.2489T > G (p.L830R)	NR/NR	*de novo*	LP	Damaging (−2.34)	Deleterious (−5.11)	Disease causing (1)	Pathological (0.99)	29.3	12.62
*CHKB* (NM_005198.5)	c.592_593delCA (p.Q198Dfs*11)	NR/NR	–	P	–	–	–	–	33	0 −0.53

*Pathogenicity predictors used: Mutation taster, DANN and FATHMM (with scores ranging from 0 to 1, where 1 is predicted the most damaging), PROVEAN (with scores equal or below −2.5 being deleterious and above −2.5 being neutral), CADD (Combined Annotation Dependent Depletion) (with scores ≥ 20 indicating that the variant is predicted to be among the 1% most deleterious substitutions in the human genome) and missense variation score (positive scores indicate intolerance to variation and negative scores are given to genes that had more variants than expected) values of gnomAD database. Allele frequency in total population (gnomAD; Genome Aggregation Database) and in Spanish population (Collaborative Spanish Variant Server; CSVS). Genetic variants have been classified following the American College of Medical Genetics and Genomics (ACMG) guidelines, using VarSome (last accessed March 24, 2021). P, pathogenic; LP, likely pathogenic; NR, not reported; M, mother; F, father; Het, heterozygous.*

### Morphological Abnormalities in the Mitochondrial Network Differ According to the Mutated Gene

**FIGURE 2:**
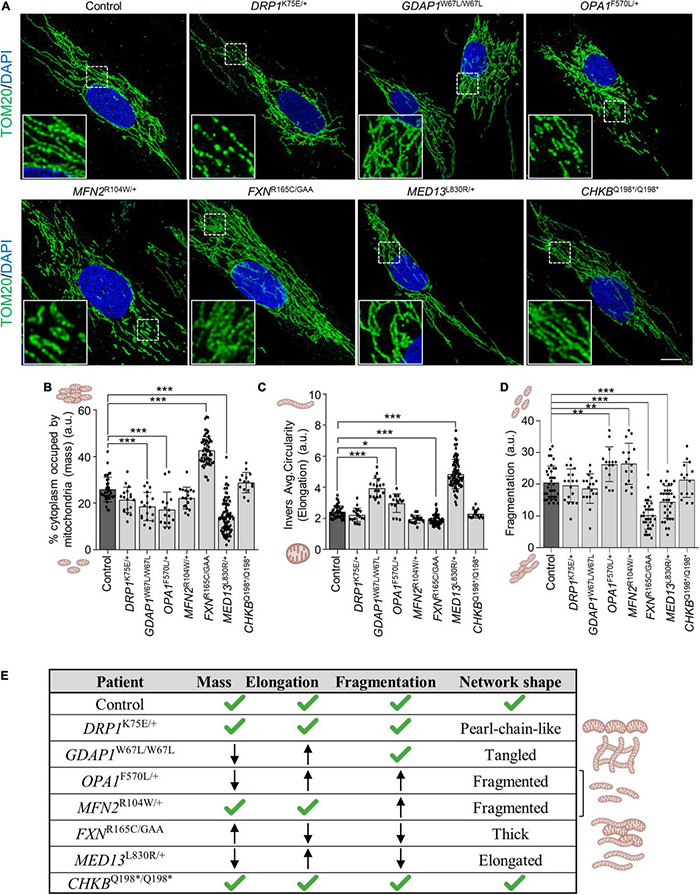
Mitochondrial network shape and quantification of mitochondrial parameters in patients’ fibroblasts. **(A)** Representative images of TOM20 mitochondrial marker immunofluorescence in control and patients’ fibroblasts. A magnification square is shown. Scale bar: 10 μm. **(B–D)** Quantification of mitochondrial mass (percentage of cytoplasm occupied by mitochondria) **(B)**, mitochondrial elongation (inverse average circularity) **(C)**, and mitochondrial network fragmentation **(D)**, in control and patients’ fibroblasts. Data represent mean ± SD and individual values are displayed as dots. One-way ANOVA followed by Dunnett’s multiple comparisons test. Three independent experiments. *P < 0.05, **P < 0.01, ***P < 0.001. a.u., arbitrary units. **(E)** Table summarizing network shape and measured mitochondrial parameters.

First, we studied in patients’ fibroblasts the mitochondrial morphology by immunostaining TOMM20 (OMM marker) and quantified mitochondrial mass, elongation and fragmentation. We observed in *DRP1*^K75E/+^ fibroblasts a peculiar network with a “pearl-chain-like” structure, which is a cellular phenotype of patients with *DRP1* variants (Nasca et al., 2016), *GDAP1*^W67L/W67L^ had a tangled network, *OPA1*^F570L/+^ and *MFN2*^R104W/+^ network were fragmented, *FXN*^R165C/GAA^ showed a thick pattern, and *MED13*^L830R/+^ showed an elongated network. In contrast, *CHKB*^Q198*/Q198*^ had a similar network when compared to control fibroblasts (Figure 2A).

Regarding quantitative parameters, the mitochondrial mass showed a significant decrease in *GDAP1*^W67L/W67L^, *OPA1*^F570L/+^, and *MED13*^L830R/+^ fibroblasts, and a significant increase in *FXN*^R165C/GAA^ (Figure 2B). The inverse average circularity showed that mitochondria were highly elongated in *GDAP1*^W67L/W67L^, *OPA1*^F570L/+^, and *MED13*^L830R/+^ fibroblasts while the opposite was found in *FXN*^R165C/GAA^ (Figure 2C). The fragmentation of the mitochondrial network was increased in *OPA1*^F570L/+^ and *MFN2*^R104W/+^ (proteins involved in mitochondrial fusion) while a significant decrement was found in *FXN*^R165C/GAA^ and *MED13*^L830R/+^ fibroblasts (Figure 2D). Taking into account all these results, we categorized the mitochondrial network shape of each patient (Figure 2D). We determined that mitochondrial networks of patients’ fibroblasts had different shapes (Figure 2E). The network was entangled in *GDAP1*^W67L/W67L^, fragmented in both *OPA1*^F570L/+^ and *MFN2*^R104W/+^, elongated in *MED13*^L830R/+^, thick in *FXN*^R165C/GAA^ and similar to control fibroblasts in *CHKB*^Q198*/Q198*^. These analyses in fibroblasts show changes in mitochondrial network morphology that are gene-specific except for *CHKB*^Q198*/Q198*^ fibroblasts.

### Patients’ Fibroblasts Show Increased Mitochondrial Oxidative Stress Without Alterations in Mitochondrial Membrane Potential (ΔΨm)

**FIGURE 3:**
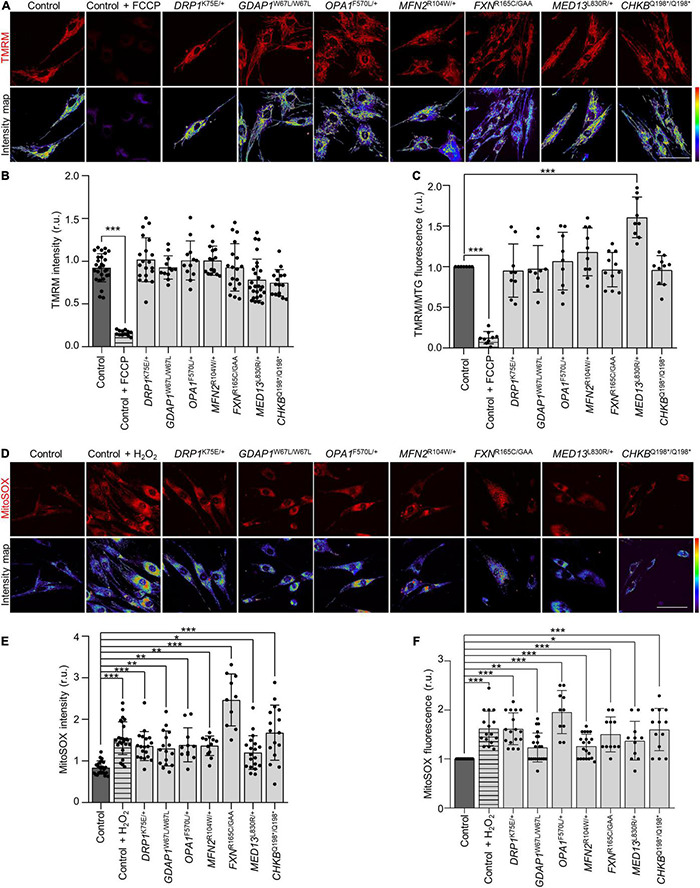
Increased mitochondrial reactive oxygen species (ROS) in patients’ fibroblasts without alterations in mitochondrial membrane potential (ΔΨ). **(A)** Representative images of *in vivo* TMRM staining in control and patients’ fibroblasts and the corresponding scattering intensity color map. As positive control cells were treated with 50 μM carbonyl cyanide-*p*-trifluoromethoxyphenylhydrazone (FCCP). Scale bar: 75 μm. **(B)** Quantification of *in vivo* TMRM intensity in control and patients’ fibroblasts. Data represent mean ± SD and individual values are displayed as dots. One-way ANOVA followed by Dunnett’s multiple comparisons test. Three independent experiments. ***P < 0.001. **(C)** Mean TMRM fluorescence intensities relative to MitoTracker Green (MTG) by flow cytometry. As a positive control, cells were treated with 50 μM FCCP. Data represent mean ± SD and individual values are displayed as dots. One sample *t*-test with multiple comparison adjustment. Three independent experiments. ***P < 0.001. **(D)** Representative images of *in vivo* MitoSOX staining in control and patients’ fibroblasts and the corresponding scattering intensity color map. As positive control cells were treated with 2 mM hydrogen peroxide (H_2_O_2_). Scale bar: 75 μm. **(E)** Quantification of *in vivo* MitoSOX intensity in control and patient fibroblasts. Data represent mean ± SD and individual values are displayed as dots. One-way ANOVA followed by Dunnett’s multiple comparisons test. Three independent experiments. *P < 0.05, **P < 0.01, ***P < 0.001. **(F)** Mean MitoSOX fluorescence intensities relative to control fibroblasts by flow cytometry. As a positive control, cells were treated with 500 μM H_2_O_2_. Data represent mean ± SD and individual values are displayed as dots. One sample *t*-test with multiple comparison adjustment. Three independent experiments. *P < 0.05, **P < 0.01, ***P < 0.001. r.u., relative units.

Since mitochondrial network dysfunction is associated with defects in mitochondria bioenergetics, and the generation of ROS (Benard et al., 2006; Kausar et al., 2018), we wondered if changes in the mitochondrial network of patients’ fibroblasts could also affect the mitochondrial membrane potential (ΔΨm) and generate ROS. The mitochondrial membrane potential was analyzed by live-cell imaging and flow cytometry using TMRM probe (Figures 3A–C). The comparison with control fibroblasts revealed significant differences only in *MED13*^L830R/+^ fibroblasts, which showed an increase in ΔΨm by flow cytometry but not in live-cell imaging. In contrast, we found mitochondrial oxidative stress in all the patients using MitoSOX probe with both technical approaches (Figures 3D–F). These results show that oxidative stress is a common cellular phenotype and a pathological marker in all patients without a decrease in ΔΨm.

### GDAP1 and MFN2 in Mitochondria–Lysosome Membrane Contact Sites of Patients’ Fibroblasts

**FIGURE 4:**
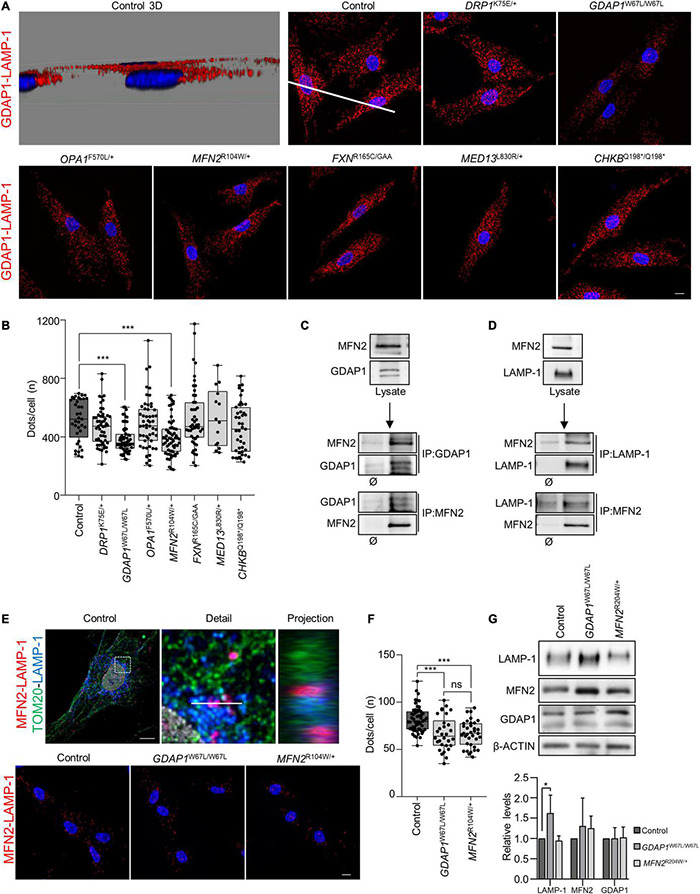
Interaction of GDAP1–MFN2 and MFN2–LAMP-1 in patients’ fibroblasts. **(A)** Representative images of proximity ligation assays (PLA) of endogenous GDAP1 and LAMP-1 interaction in control and patients’ fibroblasts. Scale bar: 10 μm. A 3D projection of the control image is shown to verify the dots localization outside the nucleus. **(B)** Quantification of the number of GDAP1-LAMP-1 dots per cell. The box plot lines correspond from the bottom of the box to the top: 25th percentile, median percentile, 75th percentile. The whiskers extend to the minimum and maximum values. Four independent experiments. Kruskal–Wallis followed by Dunn’s multiple comparisons test. ***P < 0.001. **(C)** Co-IP assay of endogenous GDAP1 and MFN2 in control fibroblasts. **(D)** Co-IP assay of endogenous MFN2 and LAMP-1 in control fibroblasts. **(E)** Proximity ligation assay with mitochondrial (TOM20, green) and lysosomes (LAMP-1, blue) co-staining in control cells, showing the dots between these organelles (*upper panel*). A deconvoluted image with an orthogonal projection is shown. Scale bar: 10 μm. Representative images of PLA of endogenous MFN2 and LAMP-1 interaction in control, *MFN2*^R104W/+^ and *GDAP1*^W67L/W67L^ fibroblasts (panel below). Scale bar: 10 μm. **(F)** Quantification of the number of MFN2-LAMP-1 dots per cell. The box plot lines correspond from the bottom of the box to the top: 25th percentile, median percentile, 75th percentile. The whiskers extend to the minimum and maximum values. Three independent experiments. Kruskal–Wallis followed by Dunn’s multiple comparisons test. ***P < 0.001; ns, not significant. **(G)** LAMP-1, MFN2 and GDAP1 relative protein levels in control, *GDAP1*^W67L/W67L^ and *MFN2*^R104W/+^ fibroblasts. Quantification is shown in the panel below. Data represent mean ± SD. One sample *t*-test with multiple comparison adjustment. Three independent experiments. *P < 0.05.

In a previous study, we have reported that GDAP1 participates in mitochondria–lysosome contacts by interacting with LAMP-1 (lysosome-associated membrane protein-1, a marker of lysosomes) (Cantarero et al., 2020). Here, we investigated the role of these contacts in all patients’ fibroblasts using proximity ligation assays (PLA). We found a significant decrease in the number of PLA dots in both *GDAP1*^W67L/W67L^ and *MFN2*^R104W/+^ fibroblasts (Figures 4A,B). Since GDAP1 and MFN2 are located in the OMM, we hypothesized that such a reduction could be caused by the interaction of these proteins. Co-immunoprecipitation assays revealed a constitutive interaction between GDAP1 and MFN2 (Figure 4C). To further investigate the relationship between these proteins, we asked if MFN2 interacts with LAMP-1 in mitochondria–lysosome contacts. Co-IP and PLA experiments revealed the constitutive interaction between MFN2 and LAMP-1, localizing PLA dots where both organelles are present (Figures 4D,E). Moreover, we found in both *GDAP1*^W67L/W67L^ and *MFN2*^R104W/+^ fibroblasts a significant reduction in the MFN2–LAMP-1 interaction (Figure 4F). Importantly, this reduction occurred without a decrease in GDAP1, MFN2, or LAMP1 expression (Figure 4G), supporting that missense variants in *GDAP1*^W67L/W67L^ and *MFN2*^R104W/+^ affect mitochondria–lysosome contacts. These results argue that MFN2 participates in mitochondria–lysosome MCSs and that the decrease in these contacts is a unique feature of patients’ fibroblasts carrying pathogenic variants in OMM genes.

### *DRP1*^K75E/+^, *GDAP1*^W67L/W67L^, *OPA1*^F570L/+^, and *FXN*^R165C/GAA^ Patients’ Fibroblasts Showed Lysosomal Abnormalities

**FIGURE 5:**
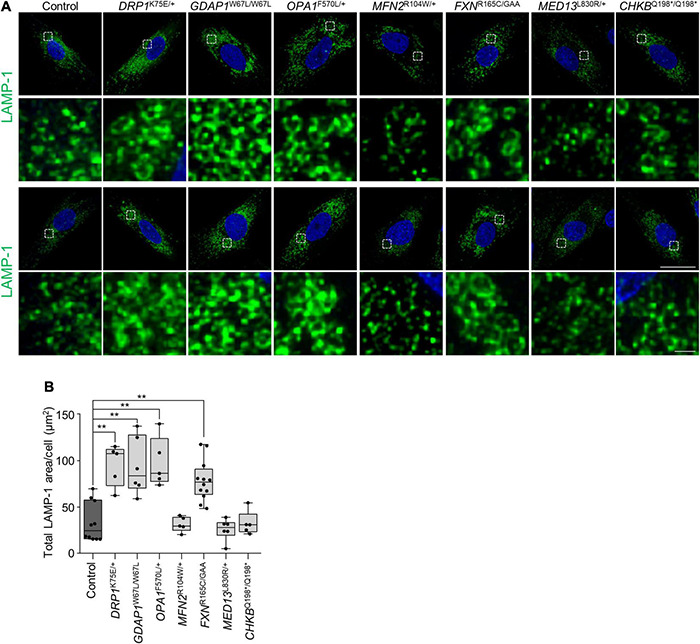
Abnormal lysosomal morphology in patients’ fibroblasts. **(A)** Two representative images of LAMP-1 lysosome marker staining in control and patients’ fibroblast. A magnification is shown in the panel below. Scale bars: 25 and 1 μm (detail). **(B)** Quantification of LAMP-1 total area per cell (μm^2^). The box plot lines correspond from the bottom of the box to the top: 25th percentile, median percentile, 75th percentile. The whiskers extend to the minimum and maximum values. Three independent experiments with at least five slides were analyzed. Kruskal–Wallis followed by Dunn’s multiple comparisons test. **P < 0.01.

Given that mitochondrial biology and mitochondria-lysosome MCSs have been reported to regulate lysosomal morphology and dynamics (Demers-Lamarche et al., 2016; Wong et al., 2018; Cantarero et al., 2020), we immunostained patients’ fibroblasts using α-LAMP-1 (Figures 5A,B). First, we analyzed *GDAP1*^W67L/W67L^ and *MFN2*^R104W/+^ fibroblasts that showed a reduction in mitochondria-lysosome MCSs (Figure 5). We found a significant increment in the lysosomal area in *GDAP1*^W67L/W67L^ but not in *MFN2*^R104W/+^ that was similar to control fibroblasts. The lysosomal area was also increased in *DRP1*^K75E/+^, *OPA1*^F570L/+^, and *FXN*^R165C/GAA^ fibroblasts. Furthermore, we observed a correlation between the lysosomal area increment and its distribution that was abnormal in *DRP1*^K75E/+^, *GDAP1*^W67L/W67L^, *OPA1*^F570L/+^, and *FXN*^R165C/GAA^ fibroblasts (Figure 5A). These results show a relationship between defects in lysosomal morphology and their distribution in fibroblasts and suggest lysosomal dysfunction.

### Starvation-Induced Autophagy Was Impaired in Patients’ Fibroblasts With Mitochondrial Membrane Protein Defects

**FIGURE 6:**
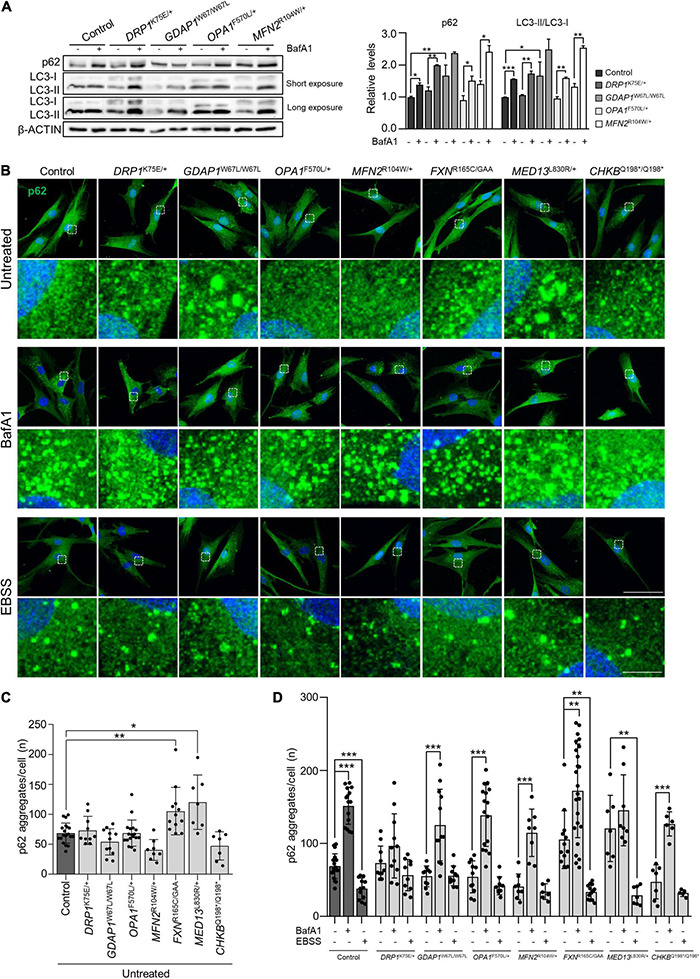
Starvation-induced autophagy was impaired in patients’ fibroblasts with mitochondrial membrane protein defects. **(A)** p62, LC3-I, and LC3-II relative protein levels in control and mitochondrial dynamic patients’ fibroblasts without treatment and after Bafilomycin A1 treatment. Quantification is shown in the right panel. Data represent mean ± SD. One-way ANOVA followed by Tukey’s multiple comparisons test. Two independent experiments. *P < 0.05, **P < 0.01, ***P < 0.001. **(B)** Representative images of autophagic flux marker p62 in untreated fibroblasts and after the inhibition (BafA1) or induction (EBSS) of autophagy. A magnification is shown in the panel below. Scale bars: 75 and 10 μm (detail). **(C)** Percentage of p62 aggregates per cell in untreated cells. Data represent mean ± SD and individual values are displayed as dots. One-way ANOVA followed by Dunnett’s multiple comparisons test. Three independent experiments with at least seven slides were analyzed. *P < 0.05, **P < 0.01. **(D)** Percentage of p62 aggregates per cell in untreated cells and after BafA1 or EBSS treatment. Data represent mean ± SD and individual values are displayed as dots. One-way ANOVA followed by Tukey’s multiple comparisons test. Three independent experiments with at least seven slides were analyzed. **P < 0.01, ***P < 0.001.

To continue examining the mitochondria-lysosome axis, we studied the autophagic flux in patients’ fibroblast with mutations in genes of mitochondrial dynamics (*DRP1*^K75E/+^, *GDAP1*^W67L/W67L^, *OPA1*^F570L/+^, and *MFN2*^R104W/+^). We performed a western blotting analysis of sequestosome-1/p62 (autophagic flux) and LC3-II/LC3-I ratio (a marker for autophagosome formation) (Bjorkoy et al., 2009) in untreated cells and after Bafilomycin A1 (BafA1) treatment (inhibits autophagosome-lysosome fusion) (Figure 6A). We found an increase of both sequestosome-1/p62 and LC3-II/LC3-I ratio in all samples except for *GDAP1*^W67L/W67L^, which showed a non-significant increase. This result in *GDAP1*^W67L/W67L^ is consistent with previous findings in *GDAP1*-deficient models (Cantarero et al., 2020). Subsequently, we analyzed in all patients the aggregates forming of sequestosome-1/p62 by immunofluorescence in cells treated with BafA1 or Earle’s balanced salt solution (EBSS), which causes starvation and promotes autophagy. In basal conditions (Figure 6B, upper panel), α-p62 immunostaining experiments revealed qualitative differences among patients regarding the size and number of p62 aggregates. Besides, *FXN*^R165C/GAA^ and *MED13*^L830R/+^ showed a significant accumulation of these aggregates (Figure 6C). After the treatments control fibroblasts showed the physiological behavior of the expected autophagic flux: BafA1 significantly increased the number of p62 aggregates per cell, while starvation had the opposite consequence with a reduction of the number of p62 aggregates (Figures 6B, medium and lower panels, and 6D). In patients’ fibroblasts, quantification of p62 aggregates resulted in four patterns of response to treatments (Figure 6D): (i) total absence of response in *DRP1*^K75E/+^, (ii) positive response to BafA1 with no response to EBSS in *GDAP1*^W67L/W67L^, *OPA1*^F570L/+^, and *MFN2*^R104W/+^, but also *CHKB*^Q198*/Q198*^, (iii) no response to BafA1 with a positive response to EBSS in *MED13*^L830R/+^ and (iv) positive response to treatments in *FXN*^R165C/GAA^, similar to the response of control cells. These results show variability among patients in terms of autophagic flux impairment and/or autophagy induction by starvation.

## Discussion

A better understanding of the pathophysiological events that underly neurogenetic disorders is increasingly necessary for the current clinical setting, characterized by searching for new biomarkers and potential pharmacological targets for the management of patients. A subset of these pathologies affects the function of mitochondria, a highly dynamic organelle, which undergoes structural and metabolic changes to respond to cellular demands. For these processes, the cell requires fusion and fission proteins, as well as other proteins that are indirectly involved in mitochondrial dynamics (Navaratnarajah et al., 2021). Indeed, the shape, size, number, distribution and interconnectivity of mitochondria are highly variable between different cell types, indicating the relevance of mitochondrial morphology for cell functioning. Here, we have investigated the mitochondrial dynamics and mitochondria-lysosome axis in fibroblasts from patients in which the mutant gene is related to the dynamics and pathophysiology of the mitochondrial network. We have been able to identify common and uncommon cellular phenotypes in these human disease cell models, and we have also found evidence of MFN2 participation in mitochondrial-lysosome MCSs, taking a further step in the knowledge of the proteins that participate in these contacts.

Regarding the morphology of the mitochondrial network, we defined specific patterns for each patient. This classification could be the basis for establishing correlations between mitochondrial network patterns and defective genes involved in mitochondrial dynamics, either directly or indirectly, that can be useful for functional studies of “variants of uncertain significance” or pharmacological screenings in fibroblasts. Other studies had associated variants in the genes studied in this work with alterations in the mitochondrial morphology. *DRP1* or *GDAP1* variants have been associated with mitochondrial fission defects thus generating hyperfused or elongated mitochondria (Niemann et al., 2005; Waterham et al., 2007; Nasca et al., 2016) while *OPA1* and *MFN2* variations cause mitochondrial network fragmentation (Del Dotto et al., 2018; Wolf et al., 2019). *FXN* and *MED13* variants have also been related to mitochondrial morphology defects (Bolinches-Amorós et al., 2014; Cooper et al., 2014). It should be mentioned that not all morphological mitochondrial disturbances can be visualized in fibroblasts as in the case of mutations in the *CHKB* gene, which are associated with giant or enlarged mitochondria concatenated in the muscles of affected subjects (Mitsuhashi et al., 2011). The *CHKB*^Q198*/Q198*^ fibroblasts did not present these disturbances nonetheless, other parameters that may be of clinical utility could be studied in this patient. For instance, we explore functional parameters related to OXPHOS, a system embedded in the inner mitochondrial membrane (Formosa and Ryan, 2018). Endeed, genetic variants in *DRP1*, *GDAP1*, *OPA1*, *MFN2*, *FXN*, *MED13*, and *CHKB* have been associated to mitochondrial ROS accumulation (Yu-Wai-Man et al., 2009; Mitsuhashi et al., 2011; Bolinches-Amorós et al., 2014; Khakhina et al., 2014; Nie et al., 2014; Cassereau et al., 2020; Longo et al., 2020). In this work, we found a significant increase in mitochondrial ROS levels in fibroblasts from all patients, suggesting a link between the abnormalities in mitochondrial network morphology and oxidative stress as previously propose (Willems et al., 2015). However, the detected increase in mitochondrial oxidative stress did not affect the mitochondrial membrane potential (ΔΨm), a key indicator of mitochondrial activity (Oliveira, 2012; Zorova et al., 2018). The exception was *MED13*^L830R/+^ fibroblasts showing a significant increase of ΔΨm that could be an adaptive response to a significantly reduced mitochondrial mass but also to the increased plasma lactic acid detected in the patient.

Beyond mitochondria, the lysosomal morphology and distribution were abnormal in *DRP1*^K75E/+^, *GDAP1*^W67L/W67L^, *OPA1*^F570L/+^, and *FXN*^R165C/GAA^ fibroblasts. These findings are in agreement with other studies that have shown that mitochondrial dysfunction (Demers-Lamarche et al., 2016) or mitochondria–lysosome MCSs defects (Wong et al., 2018; Cantarero et al., 2020) cause lysosomal alterations. Mitochondrial MCSs have gained relevance in recent years, as they constitute a physical and functional connection between organelles, with their function, and they have been associated with neurogenetic and neurodegenerative conditions (Petkovic et al., 2021; Wilson and Metzakopian, 2021). The MCSs between mitochondria and endoplasmic reticulum, MAMs, participate in autophagy (Krols et al., 2016). Specifically, the OMM supplies the membrane for autophagosome formation (Hailey et al., 2010; Hamasaki et al., 2013). We examined this process in patients’ fibroblasts and our preliminary findings suggest an altered response to autophagy induction by starvation in those cells carrying pathogenic variants in genes directly associated with mitochondrial membranes (*DRP1*, *GDAP1*, *OPA1*, *MFN2*, and *CHKB*). In addition, some of patients’ fibroblasts may have an abnormal autophagy initiation or autophagic flux. For instance, DRP1 is recruited from the cytosol in the final process of mitochondrial fission (Otera et al., 2010). The mutated protein was unable to fragment the mitochondrial network properly (pearl-chain-like pattern), which may affect the whole autophagic flux. GDAP1 and MFN2 are involved in fission and fusion, respectively, and are localized in the OMM pointing to their direct participation in early events of membrane biogenesis in MAMs. OPA1, located in the inner mitochondrial membrane and intermembrane space, is responsible for mitochondrial fusion final steps (Cipolat et al., 2004). In the case of *CHKB*, this gene encodes for a key protein in phospholipid biosynthesis and mutations in this gene could result in modified phospholipid composition of the mitochondrial membrane affecting mitochondrial function and structure (Mitsuhashi et al., 2011). Although more work is needed to decipher the autophagy defects associated with each gene, our results suggest that both the mitochondrial dynamics and the integrity of mitochondrial membranes are relevant in autophagosome formation by starvation-induced autophagy. From a clinical point of view, our findings would support the pathogenic role of autophagy impairment in some neurogenetic disorders with mitochondrial dysfunctions, which is consistent with their degenerative nature (Nixon, 2013), and could provide the rationale for developing therapeutic strategies to modulate autophagy in these conditions.

Finally, we found evidence of MFN2 localization in mitochondria–lysosome MCS and its interaction with LAMP-1. These MCSs regulate both mitochondrial network dynamics and lysosomal morphology (Wong et al., 2018). In previous studies it has shown that GDAP1 is a tether of mitochondria–lysosome MCSs, increasing the affinity of one organelle for the other (Cantarero et al., 2020). Here, we found a significant reduction of these mitochondrial MCSs in *GDAP1*^W67L/W67L^ and *MFN2*^R104W/+^ fibroblasts. Interestingly, in both cases, the pathogenic change is a missense mutation that occurs in the catalytic domain of these proteins, namely the GST-N in GDAP1 and the GTPase in MFN2 (Figure 1). The study of other missense variants located in other domains of these proteins will provide information about the structural/functional role of GDAP1 and MFN2 in mitochondria–lysosome MCSs. At the clinical level, these results suggest not only a role for MFN2 in these MCSs but also the participation of these contacts in the pathophysiology of Charcot-Marie-Tooth (CMT) neuropathy caused by mutations in *MFN2*. GDAP1 and MFN2 share some features: they are located in the OMM and participate in mitochondrial dynamics (MFN2, fusion; GDAP1, fission); regulate mitochondria-ER contacts at MAMs and both genes are linked to axonal CMT with similar clinical features. Therefore, we proposed a coordinated role of GDAP1 and MFN2 regulating MCSs with other organelles. Of note, in agreement with our proposal, it has been reported the rare coinheritance of *GDAP1* and *MFN2* pathogenic variants is associated with an accumulative effect on the observed phenotype (Kostera-Pruszczyk et al., 2014; Anghelescu et al., 2017). Indeed, it has been described the digenic inheritance in patients carrying mutations in both genes (Barreda Fierro et al., 2020).

As a final point, the fibroblasts lines of this work are from patients who attend our hospital’s outpatient clinics. These patients have been studied for being carriers of new variants in the genes associated with their disease. The use of different fibroblasts is a limitation when the objective is to compare the impact of a certain mutation on cellular phenotypes, although it has the goodness of showing the consequences on the genetic background of the patient. It would be interesting to compare different gene variants on a single genomic background by introducing the mutations by CRISPR technology into a normal fibroblast cell line.

In summary, we have identified specific and common cellular phenotypes of diseases associated with mitochondrial dynamics when compared with other disorders that impact the mitochondrial biology but not directly the mitochondrial network. The finding of MFN2 as a second protein tether between mitochondria and lysosomes along with GDAP1 highlights the relevance of mitochondrial dynamics and MCSs in the proper functioning of the organelle membrane axis in cell physiology and pathophysiology, with especial relevance in CMT neuropathy. All these results highlight the importance of translational research in fibroblasts from patients with neurogenetic diseases since they are relatively accessible models that allow us to know the pathophysiology and altered biological processes that may be of clinical interest. This knowledge can facilitate the personalized care of patients with neurogenetic diseases or the selection of patients for clinical trials with a specific cellular profile.

## Data Availability Statement

The raw data supporting the conclusion of this article will be made available by the authors, without undue reservation.

## Ethics Statement

The studies involving human participants were reviewed and approved by all procedures complied with the ethical guidelines of Sant Joan de Déu Children’s Hospital and were approved by the Clinical Research Ethics Committee under reference PIC-223-19. Written informed consent to participate in this study was provided by the participants’ legal guardian/next of kin.

## Author Contributions

FP, JH, JP, and LC contributed to the conception, design of the study, and drafted the manuscript. JP, LC, AA, AA-H, and YD-O performed the methodology, investigation, acquisition, and data analyses. DN-dB, LC-G, JE-E, CO, and AN carried out the clinical evaluation. FP and JH obtained funding. All authors performed critical revision of the manuscript for important intellectual content, and approved the final version.

## Conflict of Interest

AA was employed by HIPRA Laboratories S.A. The remaining authors declare that the research was conducted in the absence of any commercial or financial relationships that could be construed as a potential conflict of interest.

## Publisher’s Note

All claims expressed in this article are solely those of the authors and do not necessarily represent those of their affiliated organizations, or those of the publisher, the editors and the reviewers. Any product that may be evaluated in this article, or claim that may be made by its manufacturer, is not guaranteed or endorsed by the publisher.

## Footnotes

1
https://varsome.com

2
https://gnomad.broadinstitute.org

3
http://csvs.babelomics.org
